# Supplementary material for: The Probiotic BB12 Induces MicroRNAs Involved in Antigen Processing and Presentation in Porcine Monocyte-Derived Dendritic Cells
Source: Int J Mol Sci. 2020 Jan 21;21(3):687. doi: 10.3390/ijms21030687 (PMC7037397; doi:10.3390/ijms21030687)
Supplement: Supplementary file 1 [file ijms-21-00687-s001.pdf]

Supplementary Figure S1. Flow chart of in silico prediction analysis

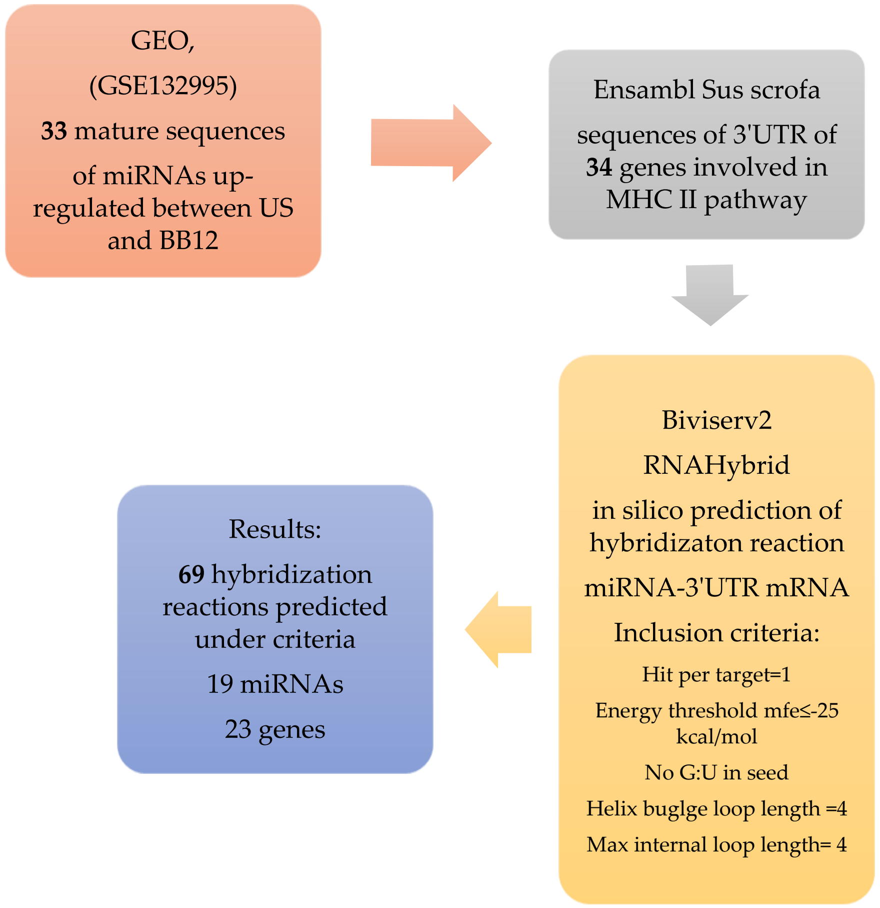

Figure S1. Flow chart of in silico prediction analysis
